# Exploring the Barriers and Facilitators in the Management of Childhood Trauma and Violence Exposure Intervention in the Vhembe District of the Limpopo Province, South Africa

**DOI:** 10.3390/children11050516

**Published:** 2024-04-25

**Authors:** Petunia Tsheole, Lufuno Makhado, Angelina Maphula, Nombulelo Veronica Sepeng

**Affiliations:** 1 Department of Psychology, Faculty of Health Sciences, University of Venda, Thohoyandou 0950, South Africa; angelina.maphula@univen.ac.za; 2 Office of the Deputy Dean Research and Postgraduate Studies, Faculty of Health Sciences, University of Venda, Thohoyandou 0950, South Africa; lufuno.makhado@univen.ac.za; 3 Department of Nursing Science, Faculty of Health Sciences, University of Pretoria, Pretoria 0082, South Africa; nombulelo.sepeng@up.ac.za

**Keywords:** childhood, healthcare workers, trauma management

## Abstract

Research has shown that barriers and facilitators in psychotherapy exhibit similarities. The authors of this study are of the view that to effectively address the difficulties encountered in psychotherapy for children and adolescents, it is crucial to consider the points of view of professionals who have firsthand encounters with children. The purpose of this study was to effectively explore barriers and facilitators in the treatment of children exposed to trauma and violence. Exploratory and descriptive methods, as components of a qualitative research design, were employed to investigate and articulate the barriers and facilitators involved in managing childhood trauma. An advertisement was used to recruit participants. It was developed and distributed to psychologists and social workers recommended by the Thohoyandou Victim Empowerment Programme. Seventeen professionals were individually interviewed using semi-structured interview schedules. The interviews were recorded, transcribed verbatim, and analysed using interpretative phenomenological analysis (IPA). The findings of the study indicated a lack of commitment from parents in honouring appointments, financial challenges, a fear of perpetrators associated with the poor reporting of incidences, professional boundaries, and referral route challenges. Familiar facilitators in the management of childhood trauma included continuous training and workshops for all people working with childhood trauma and violence, the employment of more victim advocates, and awareness campaigns. Additionally, the referral pathway for traumatised children presents logistical, psychological, and educational hurdles, underscoring the complex nature of meeting the needs of these vulnerable populations within the healthcare system. In conclusion, even while the currently available research supports the barriers and facilitators for this population, more investigation is required to examine how these factors affect treatment outcomes, particularly in community-based settings.

## 1. Introduction

Trauma to children has increased. Up to 1 billion children between the ages of 2 and 17 are estimated to have been victims of physical, sexual, or emotional abuse or neglect, according to the World Health Organisation [1]. Furthermore, it is estimated that every year, 40 million children under the age of 15 encounter violence. Children exposed to high levels of trauma and violence suffer significant harm. In South Africa (SA), 285 children lost their lives between October and December 2023, while 2707 others managed to escape situations involving assault, serious bodily harm, or attempted murder [2].

Research conducted by the Thohoyandou Victim Empowerment Programme (TVEP) indicates that the municipality of Vhembe district in Limpopo province has the highest number of trauma and violence cases against children [3]. TVEP is a prevention and support services centre located in the Vhembe district of Limpopo province that provides services to individuals impacted by sexual assault, domestic violence, and child abuse.

In 2017, TVEP reported 120 new violence cases in the Vhembe district each month. In 2018, the number increased to 200 sexual assault cases in just six months, with 60% of the victims being young children. Additionally, violence is a pervasive problem that negatively impacts the social, psychological, and physical health of a significant portion of South African children and has far-reaching effects across generations, underscoring the urgent need for prevention and treatment [4].

Everyday violent activities in Vhembe district include murder, arson of people and property, drunk driving, robbery with aggravating circumstances, grievous bodily harm, violent community protests, community mob justice, car hijacking, and robbery at residential and non-residential areas. These crimes were reported 1,393,949 times in the district and 497,237 in the Thulamela municipality alone in 2018 [5,6]. These activities are based on the researchers’ observations and the literature. According to Vhembe District Municipality [5], the Thulamela municipality is the site of almost 75% of the violent crimes reported in the Vhembe district.

Children who experience violence and trauma are vulnerable to a range of detrimental consequences that could impact their mental and physical health [7,8]. According to Hashemi et al. [9], children exposed to violence may exhibit aggressive behaviours, anxiety, and sadness. Psychological traumas such as depression, ADHD, OCD, anxiety disorders, and communication difficulties might be caused by this exposure [10]. Additionally, trauma can result in emotional, cognitive, and behavioural dysfunctions, which may lead to health risk behaviours in children, such as drug use [11]. Children who do not have access to psychotherapy treatments may develop severe mental health issues.

Many circumstances, such as historical settings, societal norms, and individual experiences, shape psychotherapy attitudes and views among South Africans. According to Van Rensburg et al. [12], traditional African healing methods are seen not only as strictly religious or spiritual pursuits but as types of psychotherapy. Additionally, Wagner [13] and Brandl [14] suggest that Africans hold fewer favourable views towards psychotherapy and psychiatric medications.

In Africa, child psychotherapy is a critical area that requires focused attention, especially for children who have endured trauma and violence. Studies by Dugmore [15] and Dugmore [16], focusing on parent-infant/child psychotherapy in South Africa, along with Nöthling [17], examining traumatised and violence-exposed teenagers in the Western Cape, underscore the importance of tailored interventions. Additionally, Bain [18], exploring group psychotherapy for high-risk mother-infant dyads in South African shelters, highlights the need for innovative approaches to address the specific challenges faced by vulnerable groups [19].

The above information indicates that children in SA, specifically in the Vhembe district, are frequently exposed to violence, and there is a likelihood that they will continue to experience trauma. It should be noted that most studies focus on adult trauma management, with very few examining childhood trauma management, particularly the impact of exposure to violence in any form. However, because of the uniqueness of a rural social setting and the evidence of the underreporting of traumatic incidents, it is concerning that many children are exposed to violence and consequently to the potential for developmental failure, trauma, and additional exposure without adequate management to mental healthcare. Furthermore, physical illnesses receive a more significant portion of South Africa’s healthcare budget than mental or psychiatric health. Research into healthcare providers with experience working with this group will, therefore, shed light on the barriers and facilitators experienced in managing children with trauma and exposure to violence.

### Aim of the Study

- The aim of the study was to explore and describe the barriers and facilitators to managing childhood trauma and exposure to violence in the Vhembe district.

## 2. Methodology

### 2.1. Study Design

The data collection process employed an exploratory-descriptive design. The primary objective of the TVEP centre is to improve the quality of care and the availability of services for children, families, and communities affected by trauma within the Vhembe district. As a component of this effort, data were collected from experts who administer evaluation and treatment. The purpose of involving psychologists and TVEP staff was to investigate firsthand experiences with interventions utilised for the treatment of trauma and exposure to violence in children, as well as potential obstacles encountered in implementing existing treatment methods.

### 2.2. Study Setting

The Vhembe district is located in the northern region of the province of Limpopo, SA. It is renowned for its diverse cultural legacy, scenic landscape, and vibrant economy. The Vhembe district’s economy is mainly agriculture, with locals relying only on this industry for their livelihood. With regard to its natural beauty and cultural heritage, the Vhembe district is important. It is the centre of many generations’ customs and traditional activities.

The research was conducted at various sites, including TVEP facilities, private practices, clinics, and hospitals offering trauma-related mental health services. The interviews were conducted over the phone, online, and in the participants’ offices, the centre, or the hospital. As a result, the participants were able to relax and feel at ease in their actual settings. In addition, phone and online interviews were used as an alternative to face-to-face interviews when participants were not available to meet in person.

### 2.3. Study Population, Recruitment, and Sampling Data Collection

The researchers employed a convenience sampling method. The study sample included healthcare professionals employed at trauma centres, including social workers, clinical psychologists, and counsellors. The purpose of using psychologists and TVEP staff was to investigate their firsthand experiences about the methods utilised and the various degrees of treatment provided for addressing trauma and exposure to violence among children. This study included employees, managers, and healthcare providers (including clinical psychologists, social workers, and registered trauma counsellors) from the public and private sectors and trauma centres. A total of 17 professionals were interviewed. The researchers ensured that a sufficient and diverse sample of participants, consisting of TVEP personnel and healthcare providers, were interviewed.

An advertisement was created to reach a diverse group of psychologists, as recommended by the TVEP. Due to the existence of privacy laws such as the Protection of Personal Information Act in South Africa [17], as well as the regulations established by the Health Professions Council of South Africa (HPCSA) and ethical guidelines followed by therapists, it was necessary to obtain the informed consent of colleagues before sharing their details. Consequently, the ad was distributed through e-mail and social media platforms. Most psychologists were employed in hospitals, and some were in private practices. Social workers and registered counsellors were stationed at the trauma centre, while others were used in the public sector. These professionals provided trauma-informed mental health services to children. Their services encompassed a broad range of evidence-based treatments.

### 2.4. Data Collection

Face-to-face, virtual, and telephonic semi-structured interviews were used to collect data among the participants of this study. The interviews were conducted with TVEP staff members, social workers, and clinical psychologists specialising in providing services to children who have been exposed to trauma and violence. The interviews with TVEP staff members offered a comprehensive understanding of the organisation.

### 2.5. Data Analysis

The researchers used six (6) steps of thematic analysis: reading digitally voice-recorded and transcribed interviews verbatim, note-taking, and identifying emerging themes, which were performed by all researchers (PT, LM, AM, and NVS) [20]. These were followed by exploring new trends, creating a table to categorise topics, and creating a final table for data analysis. Transcriptions were de-identified by replacing personal information (such as workplace or address) with pseudonyms (PT and LM). Data were encoded, clustered, and categorised into overarching themes and categories, frequently reviewed by the entire study team. The team categorised the themes into tables and labelled each with the participant number. Then, the team agreed on which themes qualified after an impartial reviewer was chosen to validate and rank the identified themes.

### 2.6. Sample Characteristics

The general workers and victim advocates did not receive trauma training and were not registered with the professional body. The general workers’ responsibility was to keep the centre clean and orderly so that victims could get clothing, food, and a warm, comfortable place to sleep or rest until they could return home, and also register and notify the appropriate authorities, including South African police forces. On the other hand, the victim advocate was responsible for emotionally supporting the victims and validating their experiences throughout the entire process, from the medical examination to the psychological assessment, as well as making follow-up calls and appointment reminders. The victim advocate had 13 years of experience, starting as a general worker and progressing through promotions to become a victim advocate.

In total, there were 17 participants; 12 of them were women and 5 were men. The sample included people in various jobs and roles. There were seven clinical psychologists, seven social workers, one victim advocate, one certified registered counsellor, and one general worker. The sample also included a variety of years of experience, especially in working with vulnerable and traumatised children. The years of experience working in those positions were 0–5 years (6 professionals), 6–10 years (10 professionals), and 11–15 years (one professional).

Six psychologists with private and public work experience were employed. One had previously worked in government and private hospitals and was employed as a lecturer during the study. The TVEP employed the victim advocate, the general worker, and the registered counsellor. The TVEP and the Department of Social Development (DSD) jointly employed the social workers. Neither the general worker nor the victim advocate had any prior experience working with trauma.

## 3. Results

### 3.1. Patient-Based Barriers

#### 3.1.1. Fear of Perpetrators

A subset of children are experiencing contextual trauma and may need a significant amount of time to initiate their removal and provide the necessary assistance due to adherence to established procedures. The participant, a social worker, indicates that the children express concerns about the potential for experiencing trauma, exposure to violence, and maltreatment.

*“Wa tseba ge e le bana ba bannyane [You know when it comes to young children] for them to go and report or tell someone in the family, …that maybe it’s your sister, my sister is abusing me, they are scared of them, of those people because they know that they will beat them when they get home and ask why did you tell those people that I beat you or I don’t give you food”*

(P1)

#### 3.1.2. Reluctant to Consult

One of the primary factors influencing patients’ successful participation in therapeutic services is their willingness to seek treatment, particularly since such services are generally voluntary. According to one social worker’s assessment, this situation can engender a sense of powerlessness and hinder patients’ ability to carry out their responsibilities effectively.

*“This client was raped for some time, and she revealed this to a friend, and the friend informed the teacher, the teacher informed the parent, and she was brought here; when she got here, she said no, I need no help, and the only person who needs help is my mom, but I could see gore [that] the child needs help, but then the child says I don’t”*

(P4)

#### 3.1.3. Financial Challenges

The psychologist highlighted factors, such as socioeconomic challenges and relocating, where the patient moves from one place to another, that might cause follow-up loss.

*“In my experience, they default due to financial issues, or due to…, maybe they have been moved to another area”*

(P14)

Socioeconomic issues are significant factors contributing to the failure to make appointments, possibly resulting in children missing sessions.

*“Majority of our people are struggling; they do not have money to come for their treatment, so that’s the challenge we are facing”*

(P3)

#### 3.1.4. Unsupportive Parents

The psychologists conveyed their frustrations regarding the insufficient commitment of parents to their children’s psychotherapy appointments. They highlighted that parents allowed different caregivers to accompany the children to the sessions, which posed a challenge in providing feedback on the child’s progress. Additionally, they expressed concerns about other situational factors that could potentially exacerbate trauma, i.e., the presence of divorced or separated parents. This can provide difficulties in obtaining feedback and comprehending the significance of therapy sessions for the child.

*“I would say sometimes it’s the commitment of parents. Some parents do not prioritise the child, so they bring it once. When they are supposed to get them for follow-up, the parent is out of town or cannot find anyone to bring the child or things like that. Another one would be if parents are separated. Sometimes, the child is with another parent, and the parent does not take matters to... It’s mostly parents”*

(P11)

The psychologists also noted that after the initial session, they propose the specific components and details of the treatment plan to the parents. The subsequent meetings involve a discussion of the topics to be covered and an examination and analysis of the assessments and their corresponding interpretations. The psychologists conveyed feelings of irritation and a sense of powerlessness in the subsequent sessions involving children. One of the challenges in managing children who have experienced trauma and violence is the issue of non-compliance with follow-up consultations. It was also emphasised that the absence of parents extends to limit intervention because most effective interventions involve parents.

*“Parents are not YOH!’’ (frustrated) compliant, especially after the first and second appointments; remember now that we will be doing the first session. After that, we will inform the parents that we will do feedback sessions and explain the assessment’s meaning. They would change guardians, miss appointments and make excuses”*

(P9)

#### 3.1.5. Unsupportive Work and School Regulations

According to the psychologist, working parents encounter challenges in bringing their children to therapy sessions due to their inability to be free from work. The participant elaborated on the challenges associated with obtaining permission from educational institutions to grant learners early departure or absences.

*“Also, parents of the children experiencing trauma would also experience challenges of getting leave of absence from work to bring children to attend psychotherapy sessions, and schools are making it difficult for children to come for follow-up sessions. Just adding on that, working parents getting time off from work is a bit difficult. If it’s a school going child, the school doesn’t want the child to be absent from school or even leave early. So, follow-up is a serious problem”*

(P10).

### 3.2. Healthcare Provider Barriers

#### 3.2.1. Professional Boundaries

The limited ability of healthcare providers to compel children to participate actively in treatment sessions is primarily due to the voluntary nature of therapy. Following professional ethics, patients must provide informed consent before medical treatment. Professionals must adhere to established protocols, which safeguard their well-being and ensure the child’s safety. The focus of this matter pertains to the child’s requirements, and even if one desires to provide further support, the profession’s ethical principles may impose certain limitations.

*“…because if you check the case, if you check the incident… The incident happened for some time, she was raped multiple times, so when she came in, she said no, I need no help, I think, she said no, she didn’t, she said I’m ok, I don’t need help, but according to the law I’m not allowed to force her. If she said no, …”*

(P4)

Another participant said, “*Our profession has rules and how we should govern ourselves and control the situation. Sometimes, that limits us because we have to do things by the book, you understand, so sometimes that is a limitation. …sometimes you wish I could proceed further, but then somehow you are limited.*” (P6)

#### 3.2.2. Delays in Referral Route

A social worker receives referrals from several sources, such as police stations, healthcare centres, hospitals, non-governmental organisations, and trauma centres. Still, they are initially directed to the district offices. From there, the referrals are forwarded to the supervisors before processing. One participant indicated that it was difficult since it required time, but the intervention and referral would be quick if the children were brought directly to them. The participants also mentioned the court’s delay and the restricted timeframe for interviewing the child and preparing the report. According to one social worker, they typically investigate incidents that occurred years ago.

*“Cases are received from the district and referred to our supervisors, who allocate them to us. The courts will expect the victim impact report and give us only two weeks to conclude the report, which is not enough time. In most cases, you might find that we only interviewed the child once and did not gather enough information”*

(P15).

The social worker expressed frustration with the defective referral process, which may take years. Additionally, the social worker said that although the child may be managing or have forgotten about the incident, the interview session could potentially re-traumatise them.

*“With those ones ‘eish’ (frustrated), the child protection unit would refer the child to our offices. You will find that I am working on a victim impact report of a child that was raped 10 years ago. You will find that a child has already forgotten or was coping, but now you will be reminding that child about something that they forgot or have found a way to cope with it, and during the interview they become emotional”*

(P17)

#### 3.2.3. Inadequate Education on Case Management and Referral

According to one social worker in a community setting, there seems to be inadequate knowledge regarding case management and the referral route, which are not aligned with the urgency of cases. Prioritising education on case management and referral routes for various departments is crucial. She mentioned that in several instances, they needed to handle the situations first but later discovered that the occurrence had been previously reported without the involvement of a professional to provide counselling for the child.

*“Firstly, referrals. Most referrals go to SAPS, where I think there might be challenges because, at times, they do not refer the cases to relevant professionals. Only after the second incident do we become aware of the first occurrence. There is a need for more education not only for communities but also for different departments involved”*

(P16).

### 3.3. Facilitators in Managing Childhood Trauma and Violence

#### 3.3.1. Continuous Training and Workshops for Healthcare Workers Working with Childhood Trauma and Exposure to Violence

The psychologists have reported a lack of recent participation in child trauma workshops or training, potentially due to difficulties in locating such opportunities or limitations within their organisations in providing such resources, particularly for those directly involved in working with children.

*“Uhmm… (long pause). No, I haven’t had training like a specific course related to trauma; I think because I am a Clinical psychologist and the scope of practice that was covered during the time of studying and internship”*

(P9)

In a general sense, the psychologists have indicated that they have not participated in workshops or any specific sessions solely dedicated to the topic of child trauma. One psychologist said she last participated in a conference on children in the juvenile justice system in 2019.

*“I remember in 2019, …before the COVID and stuff. I attended this other workshop, which focused on, you know…children overcoming trauma, but that was in 2019. Since then, I haven’t been to any other training except the one I went to recently, which was about, what was it about, hmm…, child offenders. That is the one I attended last year”*

(P14)

#### 3.3.2. Employment of More Victim Advocates

The victim advocate offered a comprehensive rationale for expanding the utilisation of victim advocates, highlighting the potential positive impacts on victims’ well-being.

*“We need more people to be employed as victim advocates, and these people will improve the lives of many victims”*

(P2)

The general worker stated that they conduct telephone follow-ups to inquire about the child’s well-being from the caregiver or parent after the child departs the centre. This method monitors the child’s behaviour after the session.

*“No, except for the victim advocate to just call the family if the child is gone back home, she must call them and ask if the child is ok. So, victim advocates must be employed in all centres”*

(P1)

#### 3.3.3. Awareness Campaigns

According to one social worker, awareness programmes are imperative to enhance children’s knowledge and understanding of trauma manifestations. Additionally, these efforts should provide support and guidance to children undergoing similar experiences by furnishing them with information about available resources for seeking assistance.

*“We can conduct campaigns to share the information with those people because sometimes you may find that what this client has experienced, there are some learners who are experiencing the same thing”*

(P4)

Similarly, another social work practitioner indicated that not only do they offer information to children in the community, but they also use the platform to educate parents on how to identify a child who has undergone trauma and exposure to violence.

*“We do campaigns for prevention and care and provide support in the community, informing the community about what to do when the child experiences trauma and how to report it. With care, …will be regarding providing a safe space for the child away from the perpetrators to ensure that they are not alone. We are there for them”*

(P15)

## 4. Discussions

The purpose of this study was to thoroughly examine the obstacles and enablers related to the care of children who have experienced trauma and violence in the Vhembe district of the Limpopo province in SA, in order to effectively address the complexities involved in trauma management for children.

Firstly, a barrier to mental health treatments appears to be a lack of understanding and awareness, which can be linked to a lack of support from parents. This barrier is related to low mental health literacy, which is common in both high- and low-income nations [21,22]. In several affluent countries, such as France, the false notion that inadequate parenting leads to ASD results in the hospitalisation of children [23]. It is evident that parents who are unaware of the need to send their children to the doctor and make the necessary arrangements to ensure that they receive treatment may be lacking in awareness of mental health issues.

Low mental health literacy among those referring patients from the courts, police, and primary care providers may also be positively correlated with referral route delays. It is also clear that specific systemic flaws can adversely affect a child’s emotional development as they learn to manage their trauma.

Secondly, fear of the perpetrators is a factor in the underreporting of child trauma cases. Traumatised children may react with a generalised fear response, perceiving a greater variety of cues as possible threats [24]. Furthermore, underreporting of abuse or violence may occur due to a fear of the perpetrator, mainly when the perpetrator is a close family member [25]. To overcome resistance from children exhibiting fear or objection, abusers may use violence or threats, furthering the vicious cycle of intimidation and anxiety [26].

The healthcare providers noted that financial difficulties caused significant rates of non-compliance and therapeutic default. Psycho-socio-cultural factors may play a role in the persistence of these false views about mental health. This decreased access is most likely made worse by financial limitations, fewer educational options, and communities with a majority of lower socioeconomic status [27,28].

Financial challenges cause rapid changes in social structures, and the stress that comes with economic instability in informal and rural settlements might exacerbate mental health issues [28]. Children may experience a complicated interaction of trauma, poverty, and depression due to social exclusion, restricted access to government services, and widespread unemployment. Additionally, according to Tsheole et al. [8] and Katz and Field [29], considering the obstacles they face, an intervention tailored specifically for African children in a rural setting is required.

The final factor regarding barriers is professional boundaries. Children who have experienced multiple traumatic events may struggle to admit when they need the help that is supposed to be provided. The voluntary character of therapy is the primary reason therapists are limited to forcing children to actively participate in treatment sessions. In SA, professional ethics requires victims, parents, or caregivers to give informed consent before medical treatment.

Enhancing people’s knowledge of the biological and behavioural causes of mental health disorders is essential to facilitating access to professional mental healthcare. Public awareness initiatives are an effective and potentially extensive means of closing the knowledge gap on mental health. Another method of filling this gap is through training [30,31]. By empowering communities in low-resource nations, mental health training may alleviate worldwide gaps in access to mental health treatments [32,33]. This training should emphasise a bottom-up strategy to expand on the information that African parents and professionals already possess.

## 5. Recommendations

Access to care may be significantly increased through interventions facilitated by parents and other non-specialist healthcare professionals [34,35]. Parental education could be made available online to circumvent the scarcity of professionals in Africa and to address the complexity of specific mental health issues [36]. There is a dire need for Trauma-Informed Care training and psychoeducation for all stakeholders involved to improve management and treatment for these vulnerable groups. If these types of training are offered at community centres and libraries, they may prove to be an affordable means of reaching individuals living in remote areas.

An extensive reassessment of the referral process from the clinic, police station, and treating physician to the community social worker, who will subsequently refer the child to the trauma centre, is necessary. This will facilitate the community social worker’s follow-up with the family and the child after their discharge from the trauma centre.

Finally, without firm political leadership and backing, sufficient funding, and the dedication of important players, the offered ideas are unlikely to be effectively implemented. Despite encouraging developments such as the South African Mental Health Care Act, efforts still need to be more dispersed and consistent.

## 6. Conclusions

The findings of this study identified barriers to resolving childhood trauma and exposure to violence to be a fear of perpetrators, financial problems, unsupportive parents, and unsupportive work and school conditions. At the same time, healthcare professionals indicated professional boundaries, referral delays, and inadequate education on case management and referral.

The facilitators, on the other hand, were identified as ongoing training and workshops for anyone dealing with childhood trauma and abuse, the employment of more victim advocates, and awareness campaigns.

While existing data confirm the barriers and facilitators for this demographic, further research is necessary to examine the impact of these variables on treatment outcomes, especially in community-based settings.

Furthermore, the challenges of the referral route for traumatised children encompass logistical, psychological, and educational dimensions, highlighting the multifaceted nature of addressing the needs of these vulnerable individuals within the healthcare system.

## Data Availability

The data presented in this study are available on request from the corresponding author. The data are not publicly available due to privacy regulations.
